# Metformin Stimulates FGF21 Expression in Primary Hepatocytes

**DOI:** 10.1155/2012/465282

**Published:** 2012-10-15

**Authors:** Eva B. Nygaard, Sara G. Vienberg, Cathrine Ørskov, Harald S. Hansen, Birgitte Andersen

**Affiliations:** ^1^Department of Pharmacology and Pharmacotherapy, University of Copenhagen, 2100 Copenhagen, Denmark; ^2^Department of Diabetes NBEs & Obesity Biology, Novo Nordisk A/S, 2760 Måløv, Denmark; ^3^Department of Incretin Biology, Hagedorn Research Institute, Novo Nordisk A/S, 2820 Gentofte, Denmark

## Abstract

Fibroblast growth factor 21 (FGF21) is a novel metabolic regulator of glucose and lipid metabolism; however, the exact mechanism of action and regulation of FGF21 is not fully understood. Metabolic status plays an important role in the regulation of FGF21, and we therefore examined whether metformin, an indirect AMPK-activator, regulates FGF21 expression in hepatocytes. FGF21 mRNA and protein expression were determined after incubation of primary cultured rat and human hepatocytes with metformin for 24 hours. To study the role of AMPK in the putative regulation of FGF21, hepatocytes were incubated with Compound C (an AMPK inhibitor) in the presence of metformin. A strong dose-dependent increase in FGF21 expression was observed in both rat and human hepatocytes treated with metformin. This effect was blocked by addition of the AMPK-inhibitor Compound C. The study shows that metformin is a potent inducer of hepatic FGF21 expression and that the effect of metformin seems to be mediated through AMPK activation. As FGF21 therapy normalizes blood glucose in animal models of type 2 diabetes, the induction of hepatic FGF21 by metformin might play an important role in metformin's antidiabetic effect.

## 1. Introduction

 Fibroblast growth factor 21 (FGF21) is a novel metabolic regulator of glucose and lipid metabolism [1–3]. FGF21 is a member of an atypical fibroblast growth factor (FGF) subfamily, which also includes FGF19 and FGF23. FGF21 is highly expressed in liver, pancreas, testis, and to a lesser extent in muscle and adipose tissue [4]. The regulation of FGF21 differs between tissues. Hepatic FGF21 is increased in response to fasting, PPAR*α*, and glucagon [1, 2], but also glucose induces the expression, which seems inconsistent with the fact that FGF21 expression is increased in response to fasting [5, 6].

 Several studies have shown increased plasma FGF21 levels in both obese and type 2 diabetic patients, which correlate with several factors involved in the metabolic syndrome, for example, nonalcoholic fatty liver disease and dyslipidemia [7–9]. In contrasts, circulating FGF21 levels were also increased in response to a prolonged starvation (7 days) in man [10]. Similarly, in rodents, plasma FGF21 is increased in animal models of obesity and type 2 diabetes (e.g., *ob/ob *mice), but also in response to prolonged fasting in mice [1, 2, 11]. Thus, these rather contrasting observations emphasize a very complex regulation of FGF21 in several tissues and indicate that FGF21 plays an important role in the state of extreme energy need or increased energy supply.

Studies in FGF21 transgenic mice have shown that FGF21 is involved in the regulation of lipolysis, fatty acid oxidation, and ketogenesis [1, 2, 12]. In animals models of type 2 diabetes treatment with recombinant FGF21 lowers blood glucose, corrects dyslipidemia, and increases energy expenditure, which makes FGF21 an attractive pharmaceutical target for treatment of type 2 diabetes [12–14].

The widely used antihyperglycemic drug metformin, lowers plasma glucose, LDL cholesterol, triglycerides and improves insulin sensitivity [15, 16]. Metformin has been shown to inhibit complex I in the respiratory chain, resulting in inhibition of ATP production [17]. This is followed by an increase in the cellular AMP : ATP ratio, that activate the AMP-activated protein kinase (AMPK), an enzyme acting as sensor of cellular energy status [16, 17]. In liver, AMPK activation decreases hepatic gluconeogenesis [18].

Here we studied the effect of metformin on the regulation of hepatic FGF21 expression in primary culture of rat and human hepatocytes. The present study shows that metformin increases the expression of FGF21, and that the effect of metformin of FGF21 expression seems to be mediated through AMPK activation.

## 2. Materials and Methods

### 2.1. Animals

All procedures involving the care and use of animals were approved by the Animal Experiments Inspectorate, Ministry of Justice, Denmark and carried out in accordance with the Novo Nordisk guidelines for the care and use of laboratory animals (J. nr. 2008/561-1455). Male Sprague-Dawley rats were obtained from Taconic, Lille Skensved, Denmark. All animals had free access to food and drinking water.

### 2.2. Isolation and Culturing of Primary Rat and Human Hepatocytes

Primary hepatocytes from *ad libitum*-fed male Sprague-Dawley rats (~200 g) were isolated by a two-step perfusion technique and cultured essentially as described by Andersen et al. [19].

Cryopreserved primary human hepatocytes were purchased from Celsis, Baltimore, USA and cultured as described above for the rat hepatocytes.

Rat and human hepatocytes were incubated in basal medium supplemented with 5.5 mM glucose, 0.1% w/v human serum albumin, and increasing concentrations of metformin (0–1500 *μ*M), 5-aminoimidazole-4-carboxamide-1-**β**-D-ribofuranoside (AICAR, 0–250 *μ*M), or 6-[4-(2-Piperidin-1-ylethoxy)-phenyl)]-3-pyridin-4-ylpyyrazolo[1,5-a] pyrimidine (Compound C, 0–8 *μ*M) (all from Sigma). Experiments were terminated after 0, 4, 6, 10, or 24 hrs by washing the hepatocytes twice with ice cold PBS (Gibco) and then placed at −80°C to obtain lysis of the hepatocytes.

### 2.3. Quantitative Real-Time PCR Analysis

Total RNA from primary rat and human hepatocytes were isolated using RNeasy mini kit (Qiagen) according to manufacturer's instructions. cDNA was synthesized using iScript reverse transcription kit (BioRad), and mRNA levels were determined by quantitative real-time PCR using a LNA probe-based system from Roche. Primers, designed using Primer3 software [20], in the FGF21 gene were: 5′CACACCGCAGTCCAGAAAG′3 (forward) and 5′GGCTTTGACACCCAGGATT′3 (reverse). Primers in the glucose-6-phosphatase (G6Pase) gene were: 5′CTCACTTTCCCCATCAGGTG′3 (forward) and 5′GAAAGTTTCAGCCACAGCAA′3 (reverse). All samples were run in triplicates and expression was calculated using the ΔΔC_T_ method. Samples were normalized to cyclophilin B expression (forward: 5′ACGTGGTTTTCGGCAAAGT3′; reverse: 5′CTTGGTGTTCTCCACCTTCC3′).

### 2.4. Protein Expression

Primary rat hepatocytes were solubilized at 0°C for 30 min in ice cold lysis buffer 10 mM Tris, pH 7.4, 100 mM NaCl, 1 mM EDTA, 1 mM EGTA, 1 mM NaF, 20 mM Na_4_P_2_O_7_, 2 mM Na_3_VO_4_, 1% Triton X-100, 10% glycerol, 0.1% SDS, 0.5% deoxycholate (all from Invitrogen), 50 *μ*L proteinase inhibitor cocktail per mL (Sigma, P-2714), and 1 mM AESBF (CalbioChem). Homogenates were cleared by centrifugation (10 min, 13.000 g, 4°C). Protein content in the supernatant was determined by the bicinchoninic acid method (Pierce Chem. Comp., Illinois, USA).

 FGF21 protein expression was measured by a FGF21 specific mouse/rat ELISA plate (BioVendor, Prague, Czech Republic) according to manufacturer's instructions.

 For immunoblotting equal amounts of protein lysate were subjected to SDS-PAGE (4–12% Bis-Tris gels) and transferred to nitrocellulose membranes. The membranes were washed with TBST (10 mM Tris HCL [pH 7.8], 50 mM NaCl, 0.05% Tween20), and blocked in startingblock (Pierce) for 1 h. Phospho-Acetyl-CoA Carboxylase (phosphoACC) 1 was detected as a single band at ~265 kDa (1 : 1000 in startingblock, Cell Signaling), and *β*-actin was detected as a single band at ~42 kDa (1 : 5000, startingblock, Santa Cruz). As secondary antibody, horseradish peroxidase conjugated antirabbit (Pierce Chem. Comp., Illinois, USA) or antimouse (Thermo Scientific) were used. The immunoblots were visualized by a LAS3000 Fujifilm (Science Imaging Scandinavia AB, Sweden) using Supersignal West Pico Chemiluminescence Substrate (Thermo Scientific). The bands for phosphoACC was normalized the beta-actin expression level and the ctrl was set to 1. 

### 2.5. AMPK Phosphorylation

The level of AMPK*α* protein phosphorylated at threonine residue 172 was determined in primary rat hepatocyte lysates, by AMPK*α* [pT172] specific ELISA (Invitrogen, CA, USA) according to manufacturer's instructions and normalized to protein content. 

### 2.6. Glycogen Accumulation

Primary rat hepatocytes were treated as above and the experiment was terminated after 24 hrs, washed with ice cold PBS, and placed at −80°C to obtain lysis of the hepatocytes. Glycogen accumulation was determined as an increase in glycogen levels and normalized to protein content. Glycogen was digested by amyloglucosidase (exo-1,4-*α*-D-glucosidase, EC. 3.2.1.3, Sigma) and glucose determined by the colorimetric glucose oxidase method as previously described by Gómez-Lechón et al. [21]. The intensity of the color reaction was measured at 340 nm using a microplate reader.

### 2.7. Caspase Activity

To assess viability of primary rat hepatocytes in presence of the different concentrations of metformin, the Apo-ONE Homogeneous Caspase-3/7 Assay was used (Promega) following manufacturer's instructions.

### 2.8. Statistical Analysis

Data are presented as means ± SEM. Differences between two groups were assessed using paired, two-tailed Student's *t*-test. Data were analyzed using GraphPad Prism version 5.0 (GraphPad Software, San Diego, CA), and the results were considered statistical significant for *P* < 0.05.

## 3. Results

**Figure 1:**
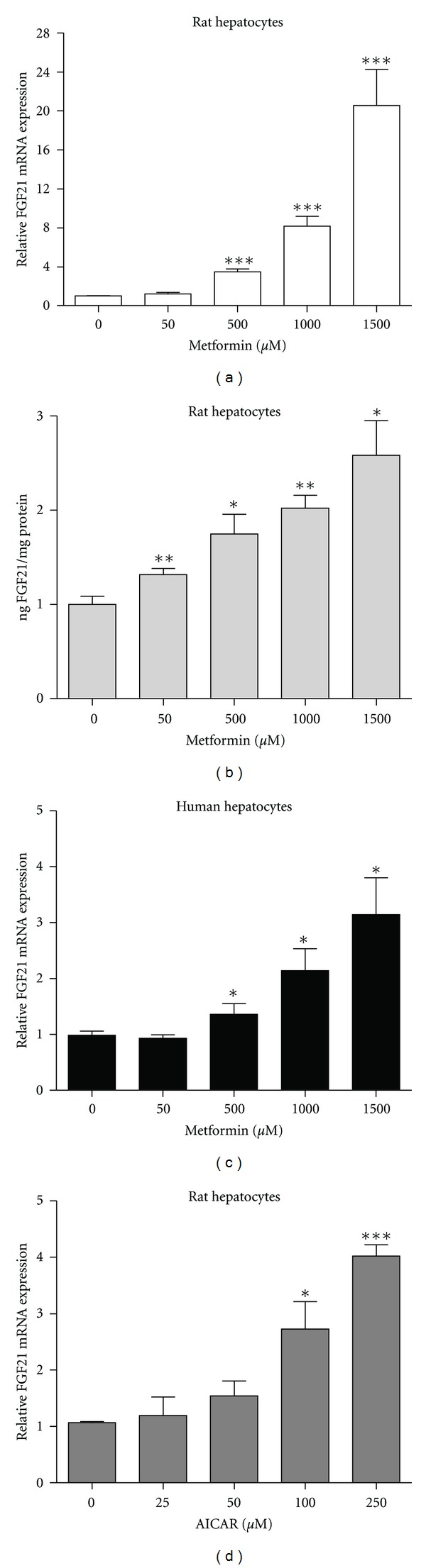
Dose-dependent effect of metformin and AICAR on FGF21 expression. (a) FGF21 mRNA and (b) protein expression in primary rat hepatocytes incubated with metformin for 24 hrs. (c) FGF21 mRNA expression in primary human hepatocytes incubated with metformin for 24 hrs. (d) FGF21 mRNA expression in primary rat hepatocytes incubated with AICAR for 24 hrs. Data are means ± SEM; *P < 0.05, **P < 0.01, ***P < 0.0001 versus nontreated hepatocytes, analyzed by paired Student's *t*-test, *n* = 4–8.

To study the effect of metformin on the regulation of hepatic FGF21, primary rat hepatocytes were incubated for 24 hrs with increasing concentrations (0–1500 *μ*M) of metformin. Metformin dose-dependently increased FGF21 mRNA expression reaching approximately ten fold at 1000 *μ*M of metformin, and FGF21 protein level were increased approximately twofold (Figures 1(a), and 1(b)). To ensure that viability of the hepatocytes after metformin-treatment was not different from untreated hepatocytes, caspase activity was determined. Same viability was found between the control and metformin-incubated hepatocytes (data not shown). No FGF21 was measured in the medium of the rat hepatocytes. The reason for this is not known, but could be due to N- or C-terminal cleavage of the FGF21 in the medium and thereby a truncated form of FGF21 that cannot be measured in the ELISA. As seen in Figure 1(c), the metformin-induced FGF21 mRNA expression was also found in primary human hepatocytes. In addition to the effect of metformin, incubation with AICAR (0–250 *μ*M), another activator of AMPK, lead to an increase in FGF21 mRNA in a dose-dependent manner (Figure 1(d)). 

**Figure 2:**
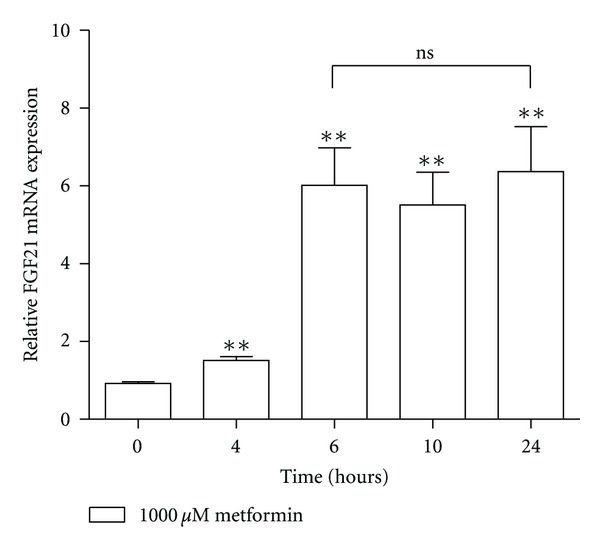
Time course of the effect of metformin on FGF21 expression. FGF21 mRNA expression in primary rat hepatocytes incubated with 1000 *μ*M metformin for 0, 4, 6, 10, and 24 hrs. Data are means ± SEM; **P < 0.01 versus non-treated hepatocytes; ns, non-significant, analyzed by paired Student's *t*-test, *n* = 5.

To study the time dependency of the effect of metformin on FGF21 expression, primary rat hepatocytes were incubated with 1000 *μ*M metformin for 0, 4, 6, 10, and 24 hrs. FGF21 mRNA expression was significantly increased after 4 hrs, and peaked at 6 hrs with no further increase after 10 and 24 hrs (Figure 2).

**Figure 3:**
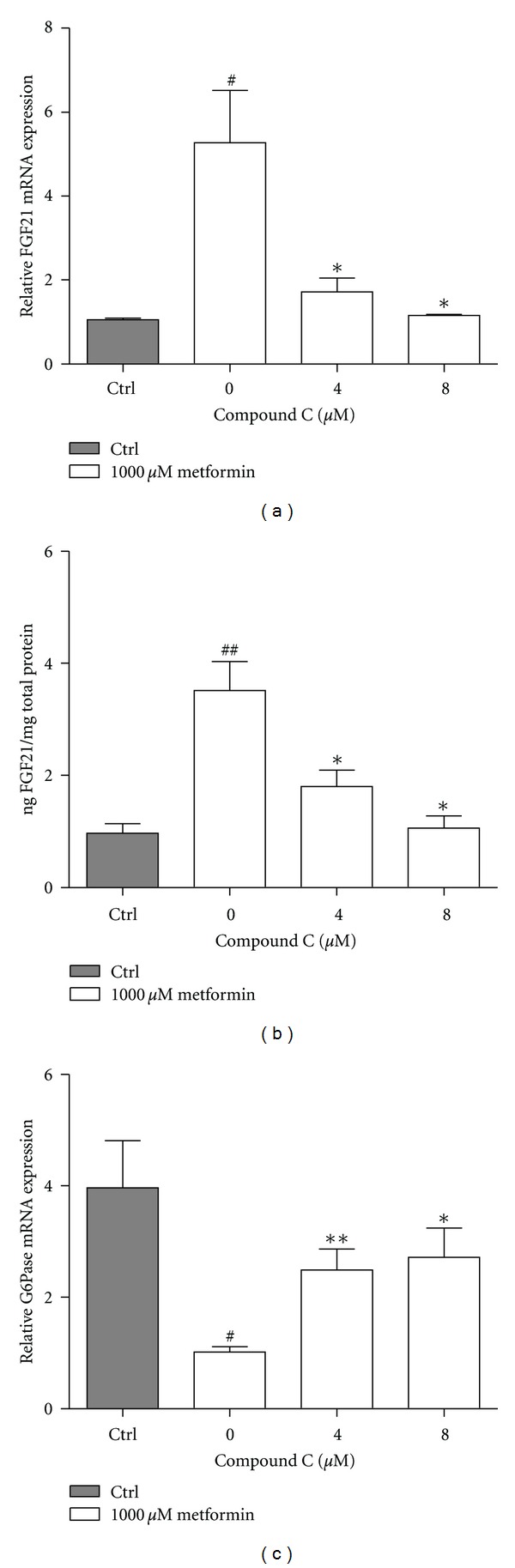
Compound C abolishes the effect of metformin on FGF21 expression. (a) FGF21 mRNA, (b) FGF21 protein, and (c) G6Pase mRNA expression in primary rat hepatocytes incubated for 24 hrs with Compound C in presence of 1000 *μ*M metformin. Data are means ± SEM; *P < 0.05, **P < 0.01 versus 0 *μ*M Compound C treated-hepatocytes, ^#^ *P* < 0.05, ^##^ *P* < 0.01 versus nontreated hepatocytes, analyzed by paired Student's *t*-test, *n* = 3–5.

Metformin has been shown to activate AMPK and to elucidate if AMPK activation was involved in the induction of FGF21 by metformin, a classical inhibitor of AMPK phosphorylation, Compound C, was applied. Primary rat hepatocytes were incubated with increasing doses of Compound C in the presence of 1000 *μ*M metformin. The effect of metformin on FGF21 mRNA and protein expression was blocked by addition of 8 *μ*M Compound C, while 4 *μ*M lowered the FGF21 expression significantly (Figures 3(a), 3(b)). Additionally, we examined the mRNA expression of G6pase as a control of metformin's action on gluconeogenic genes, known to be regulated through activation of AMPK. As expected, metformin decreased the G6Pase mRNA expression, and addition of Compound C counteracted this effect (Figure 3(c)). 

**Figure 4:**
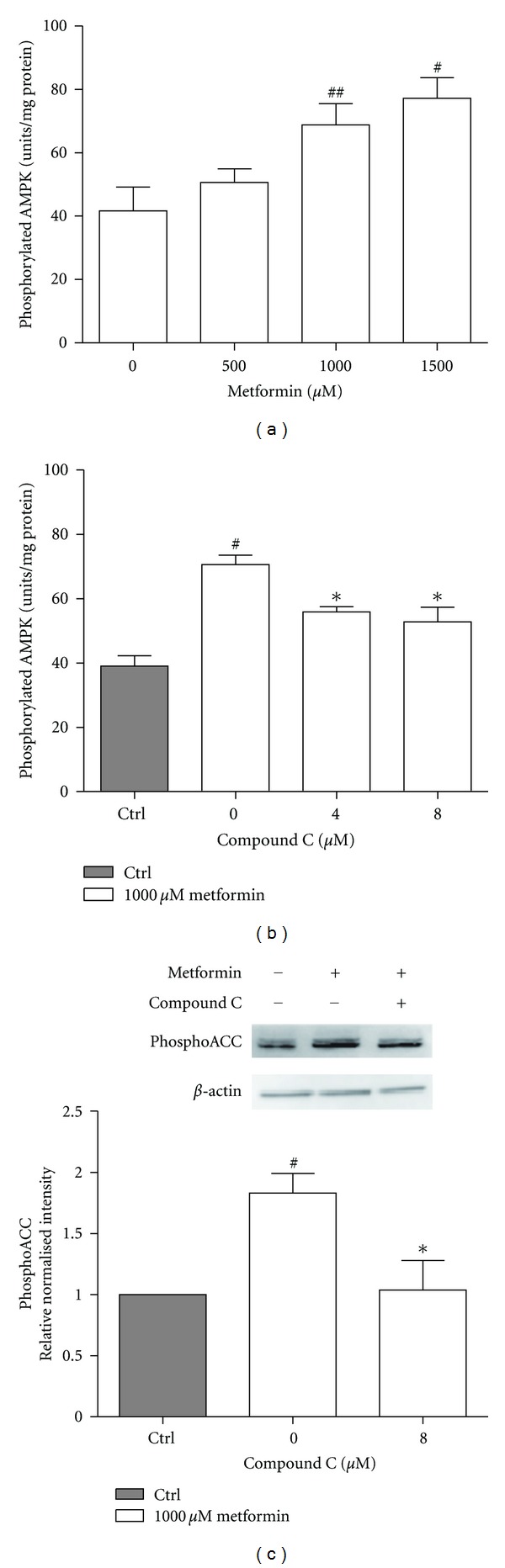
AMPK phosphorylation is stimulated by metformin. Level of (a, b) phosphorylated AMPK*α* normalized to protein content, and (c) phosphoACC detected by Western blotting, in primary rat hepatocytes. The hepatocytes were incubated for 24 hrs with (a) metformin or (b, c) Compound C in the presence of 1000 *μ*M metformin. Data are means ± SEM; ^#^ *P* < 0.05, ^##^ *P* < 0.01 versus nontreated hepatocytes, *P < 0.05 versus 0 *μ*M Compound C treated hepatocytes, analyzed by paired Student's *t*-test, *n* = 4-5.

AMPK is activated by a phosphorylation at threonine 172 (Thr172) [22]. The metformin-induced FGF21 upreguation was closely paralleled with AMPK activation, as an increase in the phosphorylation of AMPK was observed after incubating primary rat hepatocytes with metformin (Figure 4(a)) and as expected, Compound C abolished the effect of metformin on AMPK phosphorylation (Figure 4(b)). In agreement with this, the level of phosphoACC, a downstream substrate of AMPK, was increased by metformin and blocked by Compound C (Figure 4(c)). 

**Figure 5:**
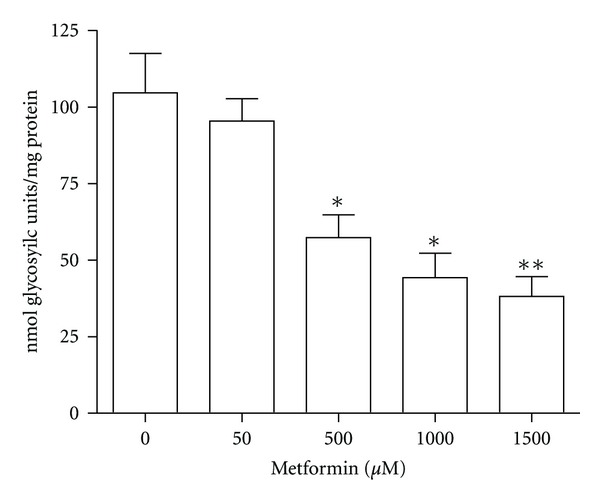
Glycogen accumulation in primary rat hepatocytes. (a) Glycogen accumulation in primary rat hepatocytes incubated with metformin for 24 hrs. Data are means ± SEM; *P < 0.05, **P < 0.01 versus non-treated hepatocytes, analyzed by paired Student's *t*-test, *n* = 5.

Treatment with metformin leads to activation of AMPK by increasing the cellular AMP : ATP ratio, and therefore low ATP level is expected (not measured). Energy demanding processes of glycogen synthesis may therefore be inhibited. The accumulated glycogen level after 24 hrs of metformin incubation of primary rat hepatocytes was therefore determined and as seen in Figure 5, a dose-dependent decrease in accumulating glycogen levels were observed with increasing concentrations of metformin.

In summary, metformin is a potent inducer of FGF21 expression in primary rat and human hepatocytes, and as the effect is blocked by addition of an AMPK inhibitor, the effect seems to be mediated through activation of AMPK. 

## 4. Discussion

The present study shows that metformin is a potent activator of FGF21 in hepatocytes *in vitro*. The concentrations of metformin used in this study are within the range of previously published studies in rat hepatocytes [17, 23], as well as within range of an *in vivo* liver exposure estimate, even though this calculation is based on several assumptions [16]. FGF21 mRNA expression was approximately 5-fold higher than the protein expression of FGF21 after incubation with metformin (1000 *μ*M). In agreement with our observations, a 24 hours fasting study on mice reported a 50-fold increase in hepatic FGF21 mRNA levels, while only a 2-fold increase in plasma FGF21 was observed [1, 2, 13]. This indicates the stability of the FGF21 protein might be relatively challenged. 

The effect of metformin on FGF21 expression in primary rat hepatocytes was blocked by incubation with Compound C. Compound C is a relatively nonspecific inhibitor of AMPK, however immunoblots of phosphoACC shows that Compound C in fact inhibits AMPK in this setup, and thus phosphorylation of the downstream substrate phosphoACC. Phosphorylation events are normally fast and the 24 hr time point may not reflect the actual picture, however, those samples were used in order to correlate with the FGF21 expression data obtained after 24 hrs. Furthermore, the 50 *μ*M metformin dose was omitted in this phosphoACC experiment, as there was no effect on FGF21 mRNA expression.

In adipocytes, FGF21 has been found to activate the AMPK-SIRT1-PGC-1*α* pathway and thereby regulate the mitochondrial oxidative capacity [24]. This suggests that the released FGF21 from the hepatocytes in our study could be the stimuli for AMPK activation; however, in the paper by Chau et al. [24], three days of incubation with 4 *μ*g/mL recombinant FGF21 is needed to activate AMPK, while the primary hepatocytes in this study only produce 2.5 ng FGF21 per mg of protein (approx.1 million hepatocytes in 1 mL of media equals 1 mg of protein), equaling a maximum of 2.5 ng/mL FGF21 for 24 hours if all produced FGF21 is released into the media. Therefore, it is very unlikely that the produced FGF21 triggers the observed activation of AMPK. Furthermore, for unknown reasons no FGF21 was found in the media of the hepatocytes after 24 hours of incubation; if this is related to the sensitivity of the FGF21 ELISA assay or fast degradation of FGF21 in the media is currently not known. 

In conclusion, both metformin and AICAR induced FGF21 expression in primary rat and human hepatocytes. Furthermore, the effect of metformin on FGF21 expression was blocked by Compound C treatment, and the specificity of this nonselective AMPK-inhibitor was confirmed by immunoblots of phosphoACC. Based on our results, we hypothesize that the regulation of FGF21 can be mediated through AMPK, which fits with the important role of AMPK in fasting [25]. This is in agreement with recent studies by Tyynismaa et al. [26] showing induction of FGF21 by decrease in ATP, caused by mitochondrial respiratory chain deficiency, and thus activation of AMPK.

The physiological and pharmacological role of FGF21 is not well understood, but the action of FGF21 seems to depend on the nutritional state of the animal. In physiology where hepatic FGF21 is increased in response to fasting [27], FGF21 might play an important role in induction of gluconeogenesis [28]; while the insulin sensitizing effect of FGF21 treatment in an insulin-resistant diabetic animal might override the physiological effect of FGF21 and thereby leads to a decrease in hepatic glucose production and blood glucose [3]. Therefore, a significant increase in hepatic FGF21 in a diabetic animal could in addition to decrease hepatic glucose production, reach adipocytes, and lead to an increase in glucose uptake [3]. Furthermore, FGF21 has also been shown to increase PPAR*γ* activity by inhibiting the degradation of PPAR*γ* by sumoylation [29]. The current study shows that metformin can regulate hepatic FGF21 *in vitro*. An examination of the effect of metformin on hepatic FGF21 expression and plasma levels *in vivo* in a diabetic mice model would be very interesting. One possible approach to examine if metformin's ability to lower blood glucose is dependent of FGF21 would be to treat mice with neutralizing FGF21 antibodies as done by Li et al. [30] in combination with metformin, but unfortunately this is without the scope of this study. However, as FGF21 is already increased in animal models of obesity and type 2 diabetes [31] it is unknown how treatment with metformin will affect hepatic FGF21 expression and FGF21 plasma level and a careful evaluation of the time course is needed. As glucose is a strong inducer of hepatic FGF21 [6], lowering of glucose by metformin will probably decrease plasma FGF21 during chronic treatment. Therefore, more studies are needed to investigate if induction of hepatic FGF21 by metformin plays a major role in metformin's antidiabetic effect. 

## Abbreviations

AICAR: 5-aminoimidazole-4-carboxamide-1-*β*-D-ribofuranoside
AMPK: AMP-activated protein kinase
FGF21: Fibroblast growth factor 21
FGFR: Fibroblast growth factor receptor
T2D: Type 2 diabetes mellitus.
